# Whole-genome sequencing and molecular characterization of Newcastle disease virus isolates from Kazakhstan

**DOI:** 10.3389/fvets.2026.1905200

**Published:** 2026-07-28

**Authors:** Ainur Nurpeisova, Makay Zheney, Perizat Akshalova, Fariza Ikramkulova, Kobeikhan Begassyl, Raikhan Nissanova, Saltanat Mamanova, Maksat Serikov, Nuray Ibraim, Nurzhan Koshkinbay, Assel Zholdasbekova, Han Sang Yoo, Markhabat Kassenov, Zhandos Abay

**Affiliations:** 1 Kazakh Scientific Research Veterinary Institute LLP, Almaty, Kazakhstan; 2 Kazakh National Agrarian Research University, Almaty, Kazakhstan; 3 College of Veterinary Medicine, Seoul National University, Seoul, Republic of Korea

**Keywords:** F gene, HN gene, Kazakhstan, lentogenic, Newcastle disease virus, phylogenetic tree, whole-genome sequencing

## Abstract

Newcastle disease virus (NDV) remains an important threat to poultry health, while complete genome data from Kazakhstan remain limited. In this study, two NDV-positive samples obtained from domestic chicken carcasses in the Almaty region were propagated in embryonated chicken eggs and characterized by diagnostic PCR/RT-PCR, infectivity assessment, whole-genome sequencing, phylogenetic analysis, and comparative molecular analysis. Both isolates replicated efficiently in embryonated eggs, reaching 9.6–9.7 log_10_ EID_50_/mL, with HA titers of 1:128–1:256 and no virus-attributable embryo mortality or marked embryo lesions. The complete consensus genomes of NDV_KZ_I and NDV_KZ_II were 15,186 bp and contained six major coding sequences arranged in the canonical 3′-N-P-M-F-HN-L-5′ order. Both isolates were closely related to the lentogenic vaccine-like strain AVIVAK-NDV-LaSota, and the F protein cleavage site motif was ^112^GRQGRL^117^, consistent with a lentogenic molecular profile. The F and HN proteins were identical between the isolates, with conserved predicted N-linked glycosylation motifs and predicted linear B-cell epitope profiles. The complete genome sequences were submitted to GenBank under submission number SUB16299204. Because the vaccination history of the source birds was unavailable and ICPI/MDT assays were not performed, the isolates should be interpreted as vaccine-like NDV detected during diagnostic investigation rather than confirmed causative agents of mortality. These data expand available NDV genomic information from Kazakhstan and support future molecular surveillance.

## 1 Introduction

Newcastle disease (ND) remains one of the most important viral diseases affecting poultry production worldwide, causing substantial economic losses in commercial and backyard poultry systems (1, 2). Newcastle disease virus (NDV), also known as avian orthoavulavirus 1, is an enveloped, negative-sense, single-stranded RNA virus with a genome of approximately 15.2 kb encoding six major proteins: N, P, M, F, HN, and L (3). Among these proteins, the fusion (F) and hemagglutinin-neuraminidase (HN) glycoproteins play key roles in viral entry, receptor interaction, membrane fusion, and pathogenicity (4–6).

The molecular epidemiology of NDV in Central Asia and neighboring regions is complex. Several genotypes and subgenotypes, including VII.2, VIg, VIIb, and VIId, have been reported in Kazakhstan and nearby countries, indicating the circulation of genetically diverse NDV lineages (7). In Kazakhstan, the detection of class II genotype VII.2 has highlighted the continued risk of virulent NDV circulation in poultry production systems (8). In neighboring regions, including the Russian Federation and Iran, subgenotype VII.1.1 has been associated with NDV evolution and cross-border spread, emphasizing the transboundary nature of NDV epidemiology (9–11). In addition, studies from Pakistan, Iran, India, and other endemic regions indicate that virulent field strains may continue to circulate even in vaccinated poultry populations (12–14).

Live attenuated vaccines, including LaSota-like and B1-like strains, are widely used for ND control. However, vaccine-like NDV strains may occasionally be detected in domestic or backyard poultry, especially when vaccination history is incomplete, undocumented, or irregular (15–18). Such findings can be difficult to interpret because vaccine-like viruses may represent recent vaccination, residual vaccine virus, environmental persistence of live vaccine strains, or limited circulation of lentogenic vaccine-derived lineages in poultry populations (16, 19, 20). Therefore, careful molecular characterization is required to distinguish vaccine-like viruses from virulent field strains and to avoid overinterpretation of diagnostic findings.

The F protein cleavage site is one of the major molecular markers used for NDV pathotype assessment. Virulent NDV strains typically possess a polybasic cleavage site, whereas lentogenic strains contain a monobasic cleavage site that restricts systemic spread (21–23). Nevertheless, molecular pathotyping based only on the F cleavage site has limitations, since additional viral proteins, including HN and polymerase-associated proteins, may contribute to replication, tissue tropism, and host adaptation (6). Whole-genome sequencing provides higher resolution than partial gene analysis and supports comparison of local isolates with vaccine strains and field strains circulating in other regions (24).

For Kazakhstan, complete genome data on NDV remain limited, although regional surveillance is needed to understand the coexistence of virulent field viruses and vaccine-like lentogenic strains (8, 25, 26). The detection of vaccine-like NDV in domestic poultry is epidemiologically relevant because it may complicate outbreak interpretation, especially when vaccination history and flock-level metadata are unavailable (16, 17, 20). Expanding whole-genome sequencing and molecular surveillance in Kazakhstan would help clarify whether vaccine-like sequences represent residual vaccine virus, undocumented vaccination, or sustained circulation of lentogenic lineages in backyard poultry systems (20).

The aim of this study was to isolate and molecularly characterize two NDV-positive samples obtained from domestic chicken carcasses submitted for diagnostic investigation in the Almaty region of Kazakhstan. The study included diagnostic RT-PCR screening, virus propagation in embryonated chicken eggs, whole-genome sequencing, phylogenetic analysis, and comparative amino acid characterization of the major NDV proteins, with particular attention to F and HN glycoproteins, predicted linear B-cell epitopes, predicted N-linked glycosylation motifs, and molecular markers associated with lentogenic vaccine-like NDV strains.

## 2 Materials and methods

### 2.1 Sample origin and virus isolation

Two NDV-positive samples analyzed in this study were obtained from domestic chicken carcasses submitted for diagnostic investigation from a private backyard flock in the Almaty region of Kazakhstan. According to the available case information, the birds showed clinical signs compatible with Newcastle disease-like infection; however, the primary cause of death could not be confirmed based on the available data. The vaccination history of the birds was not documented.

During the initial diagnostic investigation, tissue samples were screened by PCR/RT-PCR for selected avian pathogens, including avian influenza virus (AIV), fowlpox virus (FPV) and NDV. The samples were negative for AIV and FPV and positive for NDV. Tissue suspensions were prepared from pooled tracheal and lung samples, homogenized, and clarified by centrifugation. The clarified supernatants were subsequently inoculated into 10–11-day-old embryonated chicken eggs for virus isolation and propagation.

### 2.2 Virus isolation, infectivity, and hemagglutination assays

NDV-positive samples were propagated in 10–11-day-old embryonated chicken eggs by inoculation into the allantoic cavity (0.2 mL/egg). Eggs were incubated at 37 ± 0.5 °C for 72 h, monitored daily by candling, chilled at 4 °C for 16–18 h, and allantoic fluid was harvested. Virus infectivity was determined as the 50% embryo infectious dose (EID_50_/mL) using ten-fold serial dilutions in embryonated chicken eggs and calculated by the Reed–Muench method. Hemagglutination (HA) activity was determined using the standard microtiter assay with two-fold serial dilutions of allantoic fluid and a 1% chicken red blood cell suspension. The HA titer was expressed as the reciprocal of the highest dilution showing complete hemagglutination.

### 2.3 SYBR green-based one step real-time PCR

For NDV detection, viral RNA was extracted from allantoic fluid using the Qiagen Viral RNA/DNA Extraction Kit (Qiagen, Germany) according to the manufacturer’s instructions. The presence of NDV was confirmed by amplifying the F gene using a one-step RT-PCR assay with the OneTube RT-PCR SYBR kit (Evrogen, Russia). Each 20 μL reaction mixture contained 5 μL of 5 × RT-PCR Master Mix, 1 μL of reverse transcriptase, 1 μL each of the forward and reverse primers, 5 μL of RNA template, and 7 μL of nuclease-free water. Reverse transcription and PCR amplification were performed in a single tube according to the manufacturer’s instructions. Thermal cycling conditions consisted of reverse transcription at 55 °C for 15 min, followed by initial denaturation at 95 °C for 1 min and 50 amplification cycles of 95 °C for 15 s, 60 °C for 20 s, and 72 °C for 20 s. Amplification specificity was verified by melt curve analysis from 55 °C to 95 °C with 0.5 °C increments.

### 2.4 Whole-genome sequencing and bioinformatic analysis

#### 2.4.1 Library preparation

Viral RNA extracted from infected allantoic fluid was utilized for whole-genome sequencing. Sequencing libraries were prepared according to the Illumina-compatible library preparation protocol. Paired-end sequencing was subsequently performed on an Illumina platform, generating high-quality reads optimized for complete genome reconstruction of the NDV isolates.

#### 2.4.2 Read processing and consensus genome assembly

Raw sequencing reads were processed using the open-access Galaxy platform (27), where initial quality control and preprocessing were performed to remove low-quality reads and adapter sequences. Preliminary *de novo* assembly and BLASTN analysis were conducted to identify the closest complete reference genome. Based on this analysis, filtered paired-end reads were aligned to the complete genome sequence of the lentogenic Newcastle disease virus vaccine strain AVIVAK-NDV-LaSota (GenBank accession number PQ106774.1) using Bowtie2 (28). Alignment quality, mapping statistics, and sequencing depth were evaluated using SAMtools (29). Sequence variants were identified using the bcftools mpileup and bcftools call algorithms under haploid genome settings. Low-confidence variants were filtered using a quality threshold of QUAL < 20. Consensus genome sequences were generated using bcftools consensus based on the detected high-confidence nucleotide substitutions and exported in FASTA format for downstream analysis.

#### 2.4.3 Genomic characterization and phylogenetic analyses

The resulting consensus genomes were compared with nucleotide sequences available in the GenBank database using BLASTN to determine the genome length, sequence identity, and query coverage for each isolate. Coding sequences corresponding to the six major NDV genes (N, P, M, F, HN, and L) were annotated based on the closest reference genome and verified by translation into amino acid sequences. Predicted protein sequences were used for comparative amino acid analysis, including the determination of amino acid substitutions, characterization of the fusion protein cleavage site, and identification of predicted N-linked glycosylation motifs using the consensus sequence pattern N-X-S/T (where X is any amino acid except proline). Phylogenetic analysis was performed using a total of 57 F gene sequences and 32 HN gene sequences, which included the two Kazakh NDV isolates, strain I and strain II. Multiple sequence alignments were generated using the ClustalW algorithm implemented in MEGA12 (30), and phylogenetic trees were constructed using the Maximum Likelihood method with 1,000 bootstrap replicates. Finally, the amino acid sequence of the fusion protein cleavage site was analyzed to determine the molecular pathotype of the isolates.

#### 2.4.4 In silico prediction of linear B-cell epitopes and N-linked glycosylation motifs

The amino acid sequences of the F and HN proteins were used for in silico prediction of potential linear B-cell epitope regions. Prediction was performed using BepiPred-3.0 (31) in linear epitope prediction mode. Predicted regions were identified based on the top 20% predicted residues, and only continuous predicted regions of ≥5 amino acids were included in the final summary table. The predicted epitope regions were compared between NDV_KZ_I and NDV_KZ_II to assess conservation of potential antigenic regions in the two major surface glycoproteins.

Predicted N-linked glycosylation motifs were identified using the consensus sequence pattern N-X-S/T, where X represents any amino acid except proline. The epitope and glycosylation analyses were interpreted as sequence-based computational predictions and not as experimental antigenic mapping or antigenic characterization.

## 3 Results

### 3.1 Virus propagation and infectivity

The infectivity of NDV Strain I in 11-day-old ECE reached 9.6 log₁₀ EID₅₀/mL after 72 h of incubation at 37 ± 0.5 °C. Under the same conditions, the infectious titer for NDV Strain II was 9.7 log₁₀ EID₅₀/mL. Both isolates showed an embryo-based low-virulence phenotype, as no virus-attributable mortality, hemorrhages, or vascular degeneration were observed during daily candling. All embryos remained viable until the cooling stage, indicating the absence of the cytopathic effect typical of virulent strains. Successful viral replication was confirmed by HA titers in the harvested allantoic fluid, which ranged from 1:128 to 1:256.

### 3.2 Detection of NDV isolates with SYBR green-based real-time PCR

**Figure 1:**
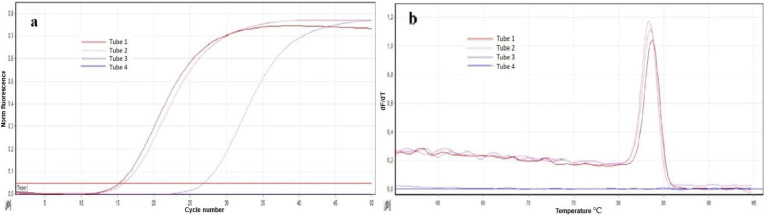
qPCR amplification and melting curve analysis of NDV F gene using selected F gene primer pairs. **(a)** Amplification curves of the F gene, **(b)** Melting curve analysis of the F gene amplicons. Tube I – Strain I; Tube 2 – Strain II, Tube 3 – positive control; Tube 4 – negative control.

SYBR Green-based real-time PCR targeting the F gene confirmed the presence of NDV in both isolates. Amplification curves showed strong fluorescence with Ct values of 15.34 for Tube 1 (Strain I) and 15.98 for Tube 2 (Strain II), indicating a high viral load. The positive control (Tube 3, RNA extracted from the LaSota strain stored at −80 °C) exhibited a Ct value of 26.94. Melt curve analysis revealed a single, sharp peak at approximately 83.5–84 °C, confirming assay specificity and absence of non-specific amplification or primer-dimers. No signal was detected in the negative control (Tube 4, nuclease-free water), validating the accuracy of the assay (Figure 1).

### 3.3 Genomic characterization, molecular assembly, and phylogenetic analysis of Kazakh NDV isolates

#### 3.3.1 Illumina sequencing data

Analysis of the Illumina raw sequencing data showed that both samples produced comparable sequencing outputs, with approximately 6.8 billion total bases and around 45 million paired-end reads per sample (Table 1). Sequencing quality was consistently high, with Q20 values exceeding 97% and Q30 values above 92%. The GC content of the raw sequencing reads ranged from 16.1 to 17.2%, reflecting the composition of the sequencing libraries prior to reference-based consensus genome reconstruction. Overall, the raw data quality and sequencing depth were sufficient for reliable downstream genome assembly and analysis.

**Table 1:** Summary of Illumina raw sequencing data for NDV-positive samples.

Sample	Total bases (bp)	Paired-end reads	GC (%)	AT (%)	Q20 (%)	Q30 (%)
Strain I	6,845,067,372	45,331,572	16.1	83.9	97.6	93.3
Strain II	6,780,314,042	44,902,742	17.2	82.9	97.4	92.9

#### 3.3.2 Complete genome assembly

Complete consensus genome sequences were obtained for both Kazakhstan NDV isolates using reference-based assembly of Illumina paired-end reads. The final genome length was 15,186 nt for both NDV_KZ_I and NDV_KZ_II, consistent with the “rule of six” characteristic of paramyxoviruses. BLASTN analysis showed that NDV_KZ_I had 100% nucleotide identity to the lentogenic vaccine-like strain AVIVAK-NDV-LaSota (GenBank accession number PQ106774.1), while NDV_KZ_II showed 99.97% identity with 100% query coverage (Table 2).

**Table 2:** Summary of complete consensus genome assembly and GenBank submission of NDV isolates.

Sample	Genome length (bp)	Query coverage (%)	Closest GenBank reference	Nucleotide identity, %	Number of CDS	GenBank submission
NDV_KZ_I	15,186	100	AVIVAK-NDV-LaSota, PQ106774.1	100.00	6	SUB16299204
NDV_KZ_II	15,186	100	AVIVAK-NDV-LaSota, PQ106774.1	99.97	6	SUB16299204

Four nucleotide differences were detected between NDV_KZ_I and NDV_KZ_II. One substitution was located in the leader/non-coding region, two substitutions were located in the P gene, and one substitution was located in the L gene. No nucleotide differences were detected in the F or HN coding sequences.

#### 3.3.3 Genome structure and gene organization

The genome organization of both Kazakhstan NDV isolates was consistent with the canonical genome structure of Avian orthoavulavirus 1. Six major coding sequences were annotated in each genome in the order 3′-N-P-M-F-HN-L-5′. The coding sequences encoded the nucleocapsid protein, phosphoprotein, matrix protein, fusion protein, hemagglutinin-neuraminidase protein, and large polymerase protein. The CDS coordinates were identical in both isolates. All coding regions started with an ATG start codon, ended with an in-frame stop codon, and showed no internal stop codons after translation (Table 3).

**Table 3:** Genome organization and coding sequences of NDV_KZ_I and NDV_KZ_II.

Gene	Product	Coordinates (nt)	CDS length (nt)	Protein length (aa)	Start codon	Stop codon
N	Nucleocapsid protein	122–1,591	1,470	489	ATG	TGA
P	Phosphoprotein	1887–3,074	1,188	395	ATG	TAA
M	Matrix protein	3,290–4,384	1,095	364	ATG	TAA
F	Fusion protein	4,544–6,205	1,662	553	ATG	TGA
HN	Hemagglutinin-neuraminidase protein	6,412–8,145	1734	577	ATG	TAG
L	Large polymerase protein	8,381–14,995	6,615	2,204	ATG	TAA

Coordinates are shown relative to the complete consensus genome sequence. CDS length includes the stop codon, whereas protein length excludes the stop codon.

#### 3.3.4 Amino acid characterization of NDV proteins

**Figure 2:**
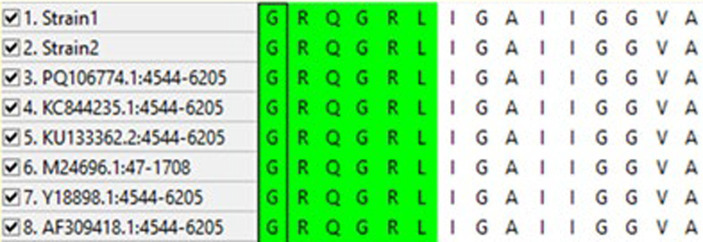
F protein cleavage site region, positions 112–117, of Kazakhstan NDV isolates compared with GenBank reference sequences. The lentogenic motif GRQGRL is highlighted in green. The Kazakhstan isolates showed 100% identity in this region to vaccine-like strains, including LaSota.

Comparative analysis of the predicted amino acid sequences demonstrated a high level of conservation between NDV_KZ_I and NDV_KZ_II. The N, M, F, and HN proteins were identical between the two isolates. Amino acid substitutions were detected only in the P and L proteins: A105V in the phosphoprotein and S778N in the large polymerase protein (Table 4). No amino acid differences were detected in the two major surface glycoproteins, F and HN. The F protein of both isolates contained the cleavage site motif ^112^GRQGRL^117^, which is characteristic of lentogenic NDV strains (Figure 2). The absence of a polybasic cleavage site is consistent with the embryo-based low-virulence phenotype observed during virus propagation.

**Table 4:** Nucleotide and amino acid differences detected between NDV_KZ_I and NDV_KZ_II.

Genome position (nt)	Gene/Region	Nucleotide change	Amino acid effect
27	Leader/non-coding region	A → T	Non-coding
2,200	P	C → T	A105V
2,609	P	T → C	Synonymous, V241V
10,713	L	G → A	S778N

Predicted N-linked glycosylation motifs were identified using the consensus sequence pattern N-X-S/T, where X is any amino acid except proline. Six predicted N-linked glycosylation motifs were detected in the F protein, at positions N85, N191, N366, N447, N471, and N541. Five predicted motifs were identified in the HN protein, at positions N119, N341, N433, N481, and N538. All predicted motifs were identical in NDV_KZ_I and NDV_KZ_II (Table 5).

**Table 5:** Predicted N-linked glycosylation motifs in F and HN proteins.

Protein	Protein length (aa)	Motif position (aa)	Motif sequence	NDV_KZ_I	NDV_KZ_II
F	553	N85	NRT	Present	Present
F	553	N191	NKT	Present	Present
F	553	N366	NTS	Present	Present
F	553	N447	NIS	Present	Present
F	553	N471	NNS	Present	Present
F	553	N541	NNT	Present	Present
HN	577	N119	NNS	Present	Present
HN	577	N341	NDT	Present	Present
HN	577	N433	NKT	Present	Present
HN	577	N481	NHT	Present	Present
HN	577	N538	NKT	Present	Present

N-linked glycosylation motifs were predicted using the N-X-S/T consensus pattern and were not experimentally validated.

#### 3.3.5 In silico prediction of linear B-cell epitopes in F and HN proteins

In silico prediction of potential linear B-cell epitope regions was performed for the F and HN proteins using BepiPred-3.0. Because the F and HN amino acid sequences were identical between NDV_KZ_I and NDV_KZ_II, the predicted epitope profiles were also identical for both isolates.

Using the top 20% predicted residues and considering only continuous regions of ≥5 amino acids, nine predicted linear B-cell epitope regions were identified in the F protein and ten regions were identified in the HN protein. In the F protein, predicted regions were located at positions 1–13, 107–116, 308–316, 350–357, 368–380, 405–415, 434–462, 492–496, and 544–549. In the HN protein, predicted regions were located at positions 5–19, 122–135, 195–202, 255–267, 321–327, 341–349, 360–372, 448–464, 494–498, and 551–555. All predicted regions were conserved in NDV_KZ_I and NDV_KZ_II (Table 6).

**Table 6:** Predicted linear B-cell epitope regions in F and HN proteins.

Protein	Protein length, aa	Predicted epitope region, aa	Peptide sequence	Length, aa	NDV_KZ_I	NDV_KZ_II
F	553	1–13	MGSRPSTKNPAPM	13	Conserved	Conserved
	553	107–116	TTSGGGRQGR	10	Conserved	Conserved
	553	308–316	VSTTRGFAS	9	Conserved	Conserved
	553	350–357	IVTFPMSP	8	Conserved	Conserved
	553	368–380	SACMYSKTEGALT	13	Conserved	Conserved
	553	405–415	PGIISQNYGEA	11	Conserved	Conserved
	553	434–462	LRLSGEFDVTYQKNISIQDSQVIITGNLD	29	Conserved	Conserved
	553	492–496	NVKLT	5	Conserved	Conserved
	553	544–549	LDQMRA	6	Conserved	Conserved
HN	577	5–19	VSQVALENDEREAKN	15	Conserved	Conserved
	577	122–135	GWGAPIHDPDYIGG	14	Conserved	Conserved
	577	195–202	GCRDHSHS	8	Conserved	Conserved
	577	255–267	TETEEEDYNSAVP	13	Conserved	Conserved
	577	321–327	KPNSPSD	7	Conserved	Conserved
	577	341–349	NDTCPDEQD	9	Conserved	Conserved
	577	360–372	KPGRFGGKRIQQA	13	Conserved	Conserved
	577	448–464	TRPGSIPCQASARCPNS	17	Conserved	Conserved
	577	494–498	GVQAR	5	Conserved	Conserved
	577	551–555	TLFGE	5	Conserved	Conserved

Linear B-cell epitope prediction was performed using BepiPred-3.0 in linear epitope prediction mode. Only continuous predicted regions of ≥5 amino acids from the top 20% predicted residues were included. The results represent computational predictions and were not experimentally validated.

#### 3.3.6 Phylogenetic analysis

**Figure 3:**
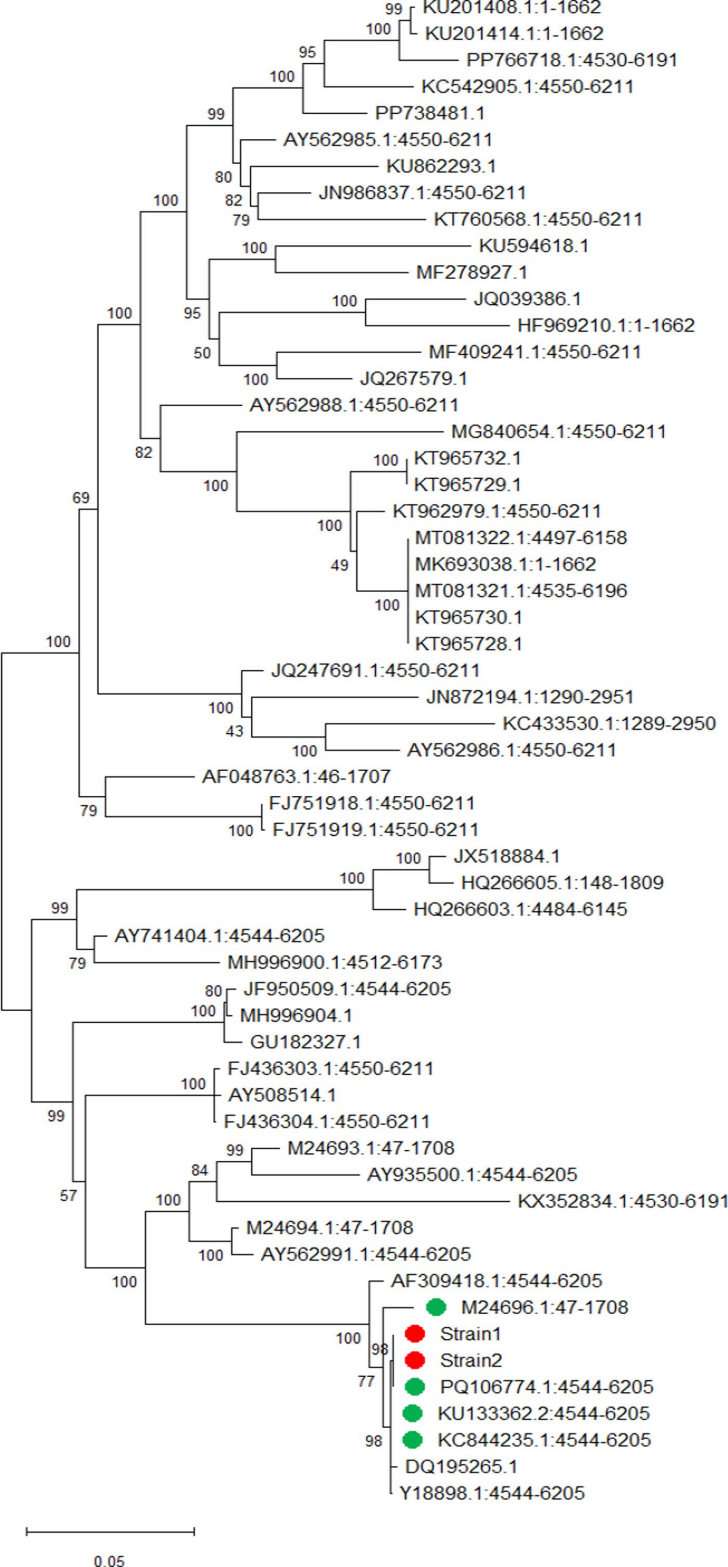
Maximum Likelihood phylogenetic tree of 57 NDV strains based on F gene sequences. The studied isolates are marked in red, and selected reference vaccine-like strains are marked in green. Bootstrap values >50% are shown at the corresponding nodes.

**Figure 4:**
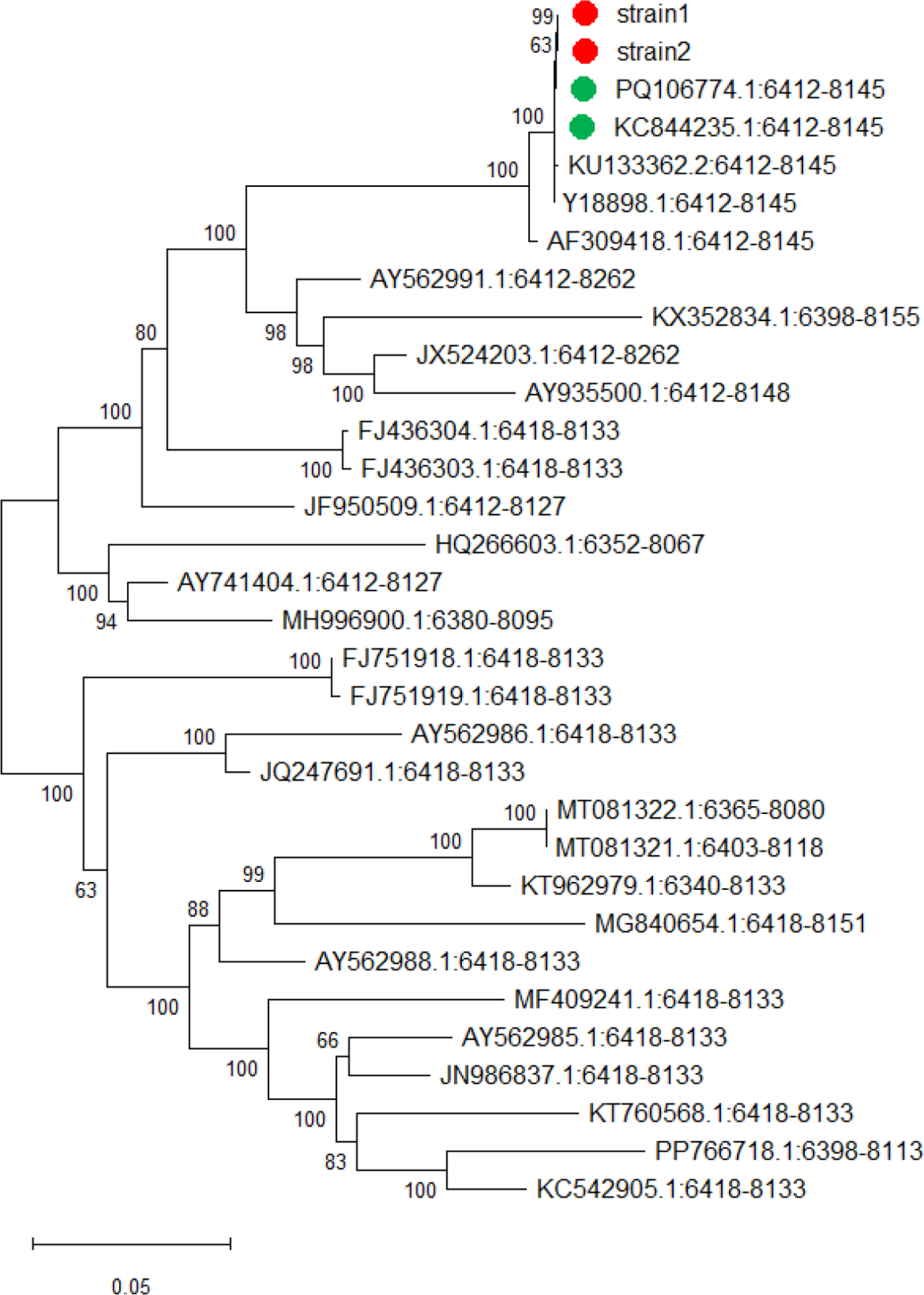
Maximum Likelihood phylogenetic tree of NDV strains based on HN gene sequences. The studied isolates, NDV_KZ_I and NDV_KZ_II, are marked with red circles. Reference sequences include GenBank accession numbers and coordinates. Bootstrap values >50% are shown at the corresponding nodes.

Phylogenetic analysis was performed using a total of 57 F gene sequences and 32 HN gene sequences, including the two Kazakhstan NDV isolates, NDV_KZ_I and NDV_KZ_II. The analysis showed that both isolates clustered with lentogenic vaccine-like NDV strains and were closely related to AVIVAK-NDV-LaSota, LaSota C5, and pigeon/Ukraine/Doneck/3/968/2007 (Figures 3, 4).

## 4 Discussion

In the present study, two NDV isolates obtained from domestic chicken carcasses in Kazakhstan were characterized using diagnostic PCR/RT-PCR screening, virus propagation in embryonated chicken eggs, infectivity assessment, whole-genome sequencing, phylogenetic analysis, and comparative amino acid analysis. The original samples were negative for avian influenza virus and fowlpox virus and positive for NDV. Both isolates replicated efficiently in embryonated chicken eggs, reaching infectious titers of 9.6–9.7 log_10_ EID_50_/mL and HA titers of 1:128–1:256. No virus-attributable embryo mortality, hemorrhages, or marked vascular degeneration were observed during incubation. These findings support an embryo-based low-virulence phenotype; however, standard pathogenicity assays such as ICPI and MDT were not performed. Therefore, the virulence interpretation should be considered molecular and embryo-based rather than a complete biological pathotyping assessment (1, 4).

The detection of NDV in carcass-derived samples should be interpreted cautiously. Although infectious virus was recovered and NDV was confirmed by RT-qPCR, the available data do not allow us to conclude that NDV was the primary cause of death of the birds. The vaccination history of the source flock was not documented, and only a limited diagnostic panel was applied. Therefore, the presence of vaccine-like NDV may reflect several possible scenarios, including recent or undocumented vaccination, residual live vaccine virus, environmental persistence of vaccine virus, limited circulation of lentogenic vaccine-like lineages, or incidental detection in birds affected by other conditions (16, 19, 20). Similar uncertainties have been described in backyard poultry systems, where incomplete flock metadata and irregular vaccination practices complicate the distinction between vaccine-related viral shedding and true field circulation (17, 18, 20).

Whole-genome sequencing provided a higher-resolution basis for interpretation of the two isolates. The final consensus genome length was 15,186 nt for both NDV_KZ_I and NDV_KZ_II, consistent with the expected genome size and the “rule of six” characteristic of paramyxoviruses (3, 24). Both genomes contained six major coding sequences arranged in the canonical order 3′-N-P-M-F-HN-L-5′. The two genomes showed extremely high similarity to the lentogenic vaccine-like strain AVIVAK-NDV-LaSota, indicating a LaSota-like genetic background. This finding is consistent with reports from neighboring and endemic regions where lentogenic vaccine-like viruses may be detected alongside virulent NDV lineages (16, 19, 32).

The regional context is important for interpreting these results. Previous studies indicate that Central Asia and neighboring regions have a complex NDV molecular epidemiology, with the coexistence of genotype VII viruses and lentogenic vaccine-like strains (7, 16). In Kazakhstan, class II genotype VII.2 has been reported, showing that virulent NDV lineages may occur in poultry production systems (8). In the Russian Federation and Iran, subgenotype VII.1.1 has been associated with recent NDV evolution and spread (9–11). Studies from Pakistan and Iran also demonstrate that virulent class II NDV strains can circulate in vaccinated poultry populations (12–14). Therefore, the detection of vaccine-like NDV in Kazakhstan should not be viewed as an isolated observation, but as part of a broader regional need to distinguish vaccine-like viruses from virulent field strains using molecular surveillance.

Phylogenetic analysis based on the F and HN genes showed that both Kazakh isolates clustered with vaccine-like lentogenic strains, including LaSota-related viruses. The F protein cleavage site motif was identified as ^112^GRQGRL^117^ in both isolates. This monobasic motif is characteristic of lentogenic NDV strains and differs from the polybasic cleavage site typically associated with velogenic and some mesogenic strains (21–23). The presence of this motif, together with the absence of embryo mortality, supports a low-virulence molecular profile. However, because ICPI and MDT were not performed, the isolates should be described as vaccine-like and lentogenic-like rather than definitively pathotyped by standard *in vivo* criteria.

Comparative amino acid analysis showed that the F and HN proteins were identical between NDV_KZ_I and NDV_KZ_II. No amino acid substitutions were detected in these major surface glycoproteins, including the F cleavage site, predicted linear B-cell epitope regions, and predicted N-linked glycosylation motifs. This conservation suggests that the two isolates share highly similar surface protein features and are unlikely to differ substantially in major sequence-based antigenic regions. In contrast, amino acid substitutions were detected only in the P protein (A105V) and L protein (S778N). Because these substitutions were located outside F and HN, their influence on antigenicity is likely limited, although their possible role in replication or host adaptation cannot be determined without functional experiments.

The *in silico* prediction of linear B-cell epitopes provided additional sequence-based characterization of the F and HN proteins. Using BepiPred-3.0, nine predicted linear B-cell epitope regions were identified in the F protein and ten regions in the HN protein. These predicted regions were fully conserved between NDV_KZ_I and NDV_KZ_II because the F and HN amino acid sequences were identical. Predicted N-linked glycosylation motifs were also conserved, with six motifs in F and five motifs in HN. Glycosylation may influence protein folding, receptor interaction, and antibody accessibility; therefore, conservation of these motifs supports the close molecular similarity of the two surface glycoproteins. Nevertheless, these findings should be interpreted as computational predictions only. No experimental antigenic mapping, cross-neutralization assay, hemagglutination inhibition assay with strain-specific antisera, or antigenic cartography was performed. Therefore, predicted epitope regions and glycosylation motifs require future experimental validation.

From an epidemiological perspective, vaccine-like NDV detection in domestic or backyard poultry has several possible implications. It may indicate recent use of live attenuated vaccines, undocumented vaccination, residual vaccine virus in the flock environment, or limited maintenance of lentogenic vaccine-like viruses in domestic birds (19, 20). In backyard systems, informal bird movement, variable biosecurity, and incomplete vaccination records may further complicate interpretation (33). Such findings are important because vaccine-like virus detection can mask or complicate recognition of co-circulating virulent NDV strains, particularly when only partial sequencing or limited diagnostic data are available. For this reason, complete genome sequencing can be useful for differentiating vaccine-related detections from field-circulating virulent viruses.

This study has several limitations. First, only two isolates were analyzed, which prevents broad conclusions about NDV diversity in Kazakhstan. Second, the vaccination history of the source birds was not documented. Third, the original diagnostic panel was limited to selected avian pathogens, and other infectious or non-infectious causes of disease could not be fully excluded. Fourth, ICPI and MDT assays were not performed. Fifth, recombination analysis, cross-neutralization assays, experimental infection studies, and antigenic mapping were not included. Finally, the predicted B-cell epitopes and N-linked glycosylation motifs were not experimentally validated. These limitations were considered when interpreting the isolates as vaccine-like NDV detected during diagnostic investigation rather than as confirmed causative agents of mortality.

Despite these limitations, the study contributes complete genome data for vaccine-like NDV isolates from Kazakhstan, a region where publicly available NDV genomic information remains limited (8, 25, 26). The results support the need for systematic molecular surveillance that combines diagnostic screening, complete genome sequencing, phylogenetic analysis, collection of flock-level metadata, and documentation of vaccination history. Future NDV surveillance in Kazakhstan should include both commercial and backyard poultry, as well as wild and synanthropic birds, because regional studies indicate that domestic and wild bird interfaces may contribute to NDV maintenance and transboundary spread (25, 34, 35). Establishing a national NDV genomic surveillance framework would help distinguish vaccine-like detections from virulent field strains, support evidence-based vaccination strategies, and improve outbreak interpretation in poultry populations (20).

Overall, the two Kazakh NDV isolates analyzed in this study showed complete genome features, F cleavage site motifs, phylogenetic placement, and surface glycoprotein conservation consistent with vaccine-like lentogenic NDV strains. The findings expand available genomic information on NDV in Kazakhstan and provide reference data for future molecular surveillance and comparative studies. At the same time, the results should be interpreted cautiously due to the limited number of isolates, lack of documented vaccination history, and absence of full biological pathotyping and experimental antigenic characterization.

## 5 Conclusion

In conclusion, two NDV isolates, NDV_KZ_I and NDV_KZ_II, recovered from domestic chicken carcasses in Kazakhstan were characterized by diagnostic PCR/RT-PCR, virus propagation in embryonated chicken eggs, whole-genome sequencing, and comparative molecular analysis. Both isolates possessed complete genomes of 15,186 bp with the canonical 3′-N-P-M-F-HN-L-5′ gene order and were closely related to lentogenic, vaccine-like LaSota-related strains. The F protein cleavage site motif ^112^GRQGRL^117^, absence of embryo mortality, and conserved F and HN protein profiles support a low-virulence, vaccine-like molecular phenotype. However, because vaccination history was unavailable and ICPI/MDT assays were not performed, the isolates should be interpreted as vaccine-like NDV detected during diagnostic investigation rather than confirmed causes of mortality. These genome data expand available NDV information from Kazakhstan and provide a useful reference for molecular surveillance and future comparative studies.

## Data Availability

The nucleotide sequence data presented in this study are deposited in the NCBI GenBank repository under accession numbers PZ619956 and PZ619957.
